# Health communication in primary health care -A case study of ICT development for health promotion

**DOI:** 10.1186/1472-6947-13-17

**Published:** 2013-01-30

**Authors:** Amina Jama Mahmud, Ewy Olander, Sara Eriksén, Bo JA Haglund

**Affiliations:** 1 School of Health Sciences, Blekinge Institute of Technology, Karlskrona, Sweden; 2 School of Computing, Blekinge Institute of Technology, Karlskrona, Sweden; 3 Department of Public Health Sciences, Karolinska Institutet, Stockholm, Sweden

**Keywords:** Health communication, Health promotion, Case study, Health promoting, Communication, EHealth, Information communication technology, Primary health care

## Abstract

**Background:**

Developing Information and Communication Technology (ICT) supported health communication in PHC could contribute to increased health literacy and empowerment, which are foundations for enabling people to increase control over their health, as a way to reduce increasing lifestyle related ill health. However, to increase the likelihood of success of implementing ICT supported health communication, it is essential to conduct a detailed analysis of the setting and context prior to the intervention. The aim of this study was to gain a better understanding of health communication for health promotion in PHC with emphasis on the implications for a planned ICT supported interactive health channel.

**Methods:**

A qualitative case study, with a multi-methods approach was applied. Field notes, document study and focus groups were used for data collection. Data was then analyzed using qualitative content analysis.

**Results:**

Health communication is an integral part of health promotion practice in PHC in this case study. However, there was a lack of consensus among health professionals on what a health promotion approach was, causing discrepancy in approaches and practices of health communication. Two themes emerged from the data analysis: Communicating health and environment for health communication. The themes represented individual and organizational factors that affected health communication practice in PHC and thus need to be taken into consideration in the development of the planned health channel.

**Conclusions:**

Health communication practiced in PHC is individual based, preventive and reactive in nature, as opposed to population based, promotive and proactive in line with a health promotion approach. The most significant challenge in developing an ICT supported health communication channel for health promotion identified in this study, is profiling a health promotion approach in PHC. Addressing health promotion values and principles in the design of ICT supported health communication channel could facilitate health communication for promoting health, i.e. ‘health promoting communication’.

## Background

Primary Health Care (PHC) has been singled out as the most suitable health care setting to meet the increasing need for health promotion interventions and to curb the rising number of chronic diseases [1-3]. A majority of people depend on health care services for health information, yet PHC is poorly equipped to provide this service [4]. Developing Information Communication Technology (ICT) supported health communication in PHC could contribute to increased health literacy and empowerment, which are foundations of health promotion and the notion of enabling people to increase control over their health and its determinants, and thereby improve their health [5,6]. It is however essential to conduct a detailed analysis of the setting and context prior to implementing an intervention in order to “avoid murky water and increase the likelihood of success” [7] (pg 506). The aim of this study was to gain a better understanding of health communication for health promotion and factors affecting such communication in a PHC setting, as a first phase in the development of an ICT supported health channel.

### Health communication

The development of health communication for promoting health has mainly taken place outside the health care services [1]. When health communication does occur within the health care services, it lacks a broad socio-ecological health promotion approach, needed to tackle lifestyle related ill health and health inequalities [8,9]. An ecological health promotion approach addresses socioeconomic and cultural factors that determine health as well as providing information and life skills to make appropriate health decisions. Such an approach includes both promoting health and preventing diseases [10], and is referred to as a health promotion approach in this paper.

Consistent with this health promotion approach, health communication in this article is defined as ‘the art and technique of informing, influencing and motivating individuals, institutional and public audiences about important health issues’ [11]. The communication adopts a participatory approach whose main aim is empowerment through dialogue and mutual learning; the process is as important as the outcome [12].

Participatory communication could facilitate collaborative learning for both provider and receiver of health communication [13]. Health communication providers can learn about receiver’s needs and preference for health communication through collaboration process; an insight that could enable them to construct health communication resources that is relevant and accessible to intended receivers. Likewise, receivers may gain more knowledge on health and health management as well as relationship between health and lifestyle through the same dialogue process. Raising health literacy of both parties is important for sustainable health care services [14].

Improving health literacy is critical to empowerment [15]. As a concept, health literacy encompasses more than transmitting health information and developing skills. It entails improving people’s access to health information and support capacity to use it effectively; in order for them to make informed choices, reduce health risks and increase quality of life [14,16]. In this light, health literacy is an important public health goal to reduce inequity [6]. The Ottawa Charter identified creation of supportive environment, developing of personal skills and reorienting health services as important action areas [17]. These action areas are incorporated in the Swedish Public Health policy [18], whose overarching goal is ‘to create societal conditions to ensure good health, on equal terms, for the entire population’. To achieve this, eleven goal areas have been identified, two of which are; to enable citizen participation in social and health care services; and to re-orient health care services into a more health promoting health services [18].

### ICT- mediated health communication

ICT mediated health communication media, with internet at the forefront, are increasingly becoming an accepted strategy for communicating health. Internet’s flexibility and accessibility through different channels makes it an ideal platform for communicating health [19,20]. Health channel in this paper is defined as a mode of transmission that enables messages to be exchanged between “senders” and “receivers.” In the context of internet, senders of the communication may have to contend with participants who engage, contest, reframe and deepen the messages in the communication process. This may take place either in an on-going dialogue in real-time or via other feedback avenue [21]. Implementation of ICT for health communication or aspects of ICT in health communication, as in eHealth applications, is essential to meet growing demands for cost-effective, appropriate and individually tailored health care as well as to increase accessibility to health services [22], improve population health outcomes and to achieve health equity [19]. Yet the implementation of ICT supported health communication for health promotion within health care services has been slow in uptake [8,19]. Criticism has been leveled at the existing ICT mediated health communication in health care as it is perceived to be predominantly individual based and pro-medicine in its approach [4,23], lacking a holistic approach and ability to address determinants of health [22]. Thus there is a need to rethink health promotion in planning for ICT mediated health communication [8,22] for a holistic approach in conceptualization and design of ICT systems in health care [24]. Innovative ways to design ICT systems in health care can contribute to individual wellbeing and quality of life, and achieve improved public health and sustainable e-services in general [25].

In the light of the challenges facing PHCs and opportunities presented by ICT in health care services outlined in the background, there is need to conduct a feasibility study prior to implementation of a new ICT supported health communication tool; in order to situate practice in its context and increase the likelihood of success [7]. Implementation of ICT is expensive, time consuming and often quickly outdated [8,26]. In order to develop sustainable ICT systems that fulfill health promotion goals in PHC, there is a need for both the system developers and health personnel to understand what functions the system is supposed to fulfill and the contexts in which it is to function [27]. This need informs the aim of this study which is to gain a better understanding of health communication for health promotion and factors affecting such communication in a PHC setting. This study has the potential to guide researchers and PHC managers in future feasibility studies and/or the implementation of ICT systems.

### Study setting

The study was conducted within a county council owned PHC and its health promotion center ‘*Hälsotorg*’ in the southeast of Sweden which provides health services to approximately 10,500 inhabitants. The PHC center houses several units: General Practitioner (GP) and District Nurse (DN) consultations services, Child Health Services (CHS), *Hälsotorg,* Pharmacy, Dental and Psychiatric Clinic.

The *Hälsotorg* was partly owned and managed by the PHC. *Hälsotorg* emerged in several county councils in the 1990’s as a collaboration between the then, state owned, pharmaceutical company and PHC in a bid to increase health promotion within the PHC services [28]. According to local evaluation reports, the concept and ambitions of *Hälsotorg* were appreciated by health personnel as well as visitors [29]. As it contributed to the alliance building with other actors working in the field of health, opened up PHC to the non patient segment of the society and thereby increasing citizens’ accessibility to and participation in health care as stipulated by the national public health policy [18]. This makes PHC a natural entry point for reorientation of health care towards a more health-promoting health services as proposed by the World Health Organization (WHO) [17,30] and the Swedish National Public Health Policy [18].

To improve accessibility to health promotion initiatives for the local community, a research and development project entitled ‘Virtual *Hälsotorg*’ (VHT) was initiated to make *Hälsotorg* activities more accessible to the local community through an internet supported interactive health channel. The main objective of the VHT project was to develop an interactive digital health channel for health promotion, a virtual “meeting place” for health issues between community members and health care personnel in PHC. According to the project goals, VHT channel was to be specifically adapted to the socio-cultural context of PHC and the local community. The VHT project was part of an EU funded research and development project exploring how ICT can be used to increase citizens’ accessibility to and participation in health care, and development of health care services.

## Methods

### Study design

The Virtual Hälsotorg (VHT) research project adopted a Participatory Action Research (PAR) approach [31]. A model, entitled Spiral Technology Action Research (STAR) [27], was used to guide the design process. The STAR model combines health promotion and social theories, PAR approach, critical pedagogy and ICT systems design approaches using rapid cycle of change strategies [27]. The iterative nature of the STAR model allowed continuous feedback and dialogue between partners in the research project which resulted in action/improvement of the product thereby making it a tangible method for realize the PAR approach of the project. The STAR model consists of five developmental cycles entitled; *Listen, Plan, Do, Study and Act*. For the VHT project, these cycles were combined to form three developmental phases; phase 1; Listen, phase 2; Plan and Do, phase 3; Study and Act. This article covers the first phase *Listen*; which entails ‘scanning the setting’. This article had a dual purpose. First, to familiarize with the setting for the intervention. Second, to assess health communication needs and identify subject’s interaction with technology. The goal of this phase in the VHT project was to ensure that the development of the system was guided by the users, both health professionals and the local population, needs as expressed by them [27].

A qualitative exploratory case study [32] methodology with multiple data collection methods; field study with participatory observations, document studies and focus groups were applied in the study to facilitate a holistic view of health communication practiced at *Hälsotorg* and PHC (Table 1). PAR approach, provided possibilities to understand individual and organizational factors as well as the relationships between these factors [32,33]. Since the boundary between *Hälsotorg* and its context (PHC) were not clearly evident, the whole context was treated as a single case study [32]. The case and unit of analysis was the phenomenon ‘health communication’ in the context of PHC in general and *Hälsotorg* in particular. According to Yin, use of multiple sources of evidence allows the investigator to address a broader range of issues comprehensively thereby contributing to convincing and accurate findings or conclusions [32] hence increasing credibility and trustworthiness of the results [33].

**Table 1:** Summary of data description, sources and methods used for data collection

**Data source**	**Sample description**	**Data collection methods**
**Field study**	A total of 251 visitors were registered during the 3 week period of field studies.	Participatory observations with a field manual to guide data collection under 3 months; 2 days a week in 2008–2009.
		A manual was used to guide data collection
**Documents**	One National Public Health Policy 2007/08:110	National policy documents identified and attained during previous study in the same project.
	One National pharmacy (Apotekets AB) Action Plan 2002.	County council documents retrieved through manual and internet searches through County Council website. using the terms “Hälsotorg”,
	Three County Council plans; 2007–2009, 2008–2010, 2009–2011	“Health promotion AND Sweden”, “Primary Health Care” and “Policy” Combinations of these terms were also used.
	One Hälsotorg evaluation report 2008	Monthly Hälsotorg reports kept by personnel under the field study period documenting
	Three Hälsotorg network Meeting	Hälsotorg activities and visitor’ statistics
	protocols 2008–2009	Health information materials at Hälsotorg
	Four Monthly reports covering the period of field study from the four Hälsotorgs in the region 2008	collected during the field study
	Printed materials on lifestyle health problems from Hälsotorg.	
	Information on Hälsotorg activitities.	
**Focus groups**	Total (N=30) persons took part in 5 groups of 3–9 persons/group	Focus group discussions, using semi-structured interview guide.
	**Group 1 and 2**: DN from PHC (n=9)	
	**Group 3**: Hälsotorg network group consisting of 6	
	(3 pharmacists, 3 DN’s) Hälsotorg personnel from the other three Hälsotorgs in the region , 1 PHC manager, 1 Regional public health strategist, 1 Psychiatry clinic manager and 1 Dental clinic manager (n=10)	
	**Group 4:** Immigrants at a Swedish language instructions class (n=8; 6 women, 2 women), ages 26–50. Length of stay in Sweden 6 months – 8 years	
	**Group 5:** Hälsotorg Personnel in PHC setting of the case study (n=3)	

### Case description

*Hälsotorg* in this study was managed by health professionals from the PHC and the Pharmacy. It offered a range of health promotion activities including health information in print and electronic media, individual health counseling on life style related health problems like stress, physical inactivity, overweight and chronic diseases such as hypertension and diabetes. It also offered group activities such as: open public lectures, ‘power walking’ and aerobics for people with physical disabilities. A customer computer placed at the *Hälsotorg;* provided access to free, trustworthy internet-based health information sites and self-administered lifestyle tests. All activities were open to all citizens free of charge.

The term ‘visitor’ was used to describe all who visited *Hälsotorg,* regardless of how or why they came, in contrast to ‘patients’ in other PHC units. *Hälsotorg* personnel did not have an obligation to document visitors in the electronic patient record, thus all visitors had the right to be anonymous. *Hälsotorg* had two types of clientele; visitors, who visited of their own accord and visitors who came on referral from GP, DN or CHS.

The case was expanded to include experiences of personnel from the other three *Hälsotorg* in the region to get a broader perspective of health promotion services offered and to solicit input on the content and development of a VHT model usable in all county council owned PHC in the region. The GP and DN consultations services, CHS and *Hälsotorg* belong to the same organization and will henceforth be referred to collectively as ‘PHC’ in this paper, likewise, personnel from respective units will be referred to as ‘health personnel’, unless the need to separate them arises.

### Fields study

To familiarize with the setting for the intervention, find and assess needs, and identify how subjects interacted with technology, a field study was conducted under a period of three months, twice a week, in 2008–2009. AJM took part in *Hälsotorg* activities and staff meetings in the PHC, collecting data using participatory observations [33]. A total of 251 people visited the *Hälsotorg* during the period of the field study, some of whom took part in the informal interviews which formed part of the field notes.

Participatory observation as a method contributed to a better understanding of the context, its actors and their interrelations. Thereby a nuanced understanding of the context as a basis for understanding data collected through other methods such as focus groups and document studies [33]. Furthermore, findings from the participatory observations were used to identify key actors (study sample) and to design questions for the focus group. Participatory observation was useful as expression of needs, especially for technology based resources, is often tacit and hard to deduce for the majority of the people [31,34].

A field study manual covering; activities conducted at *Hälsotorg*, participants and reason for participation. The manual also focused on how health communication was framed and communicated as well as tools and strategies used to communicate health. The interaction between health personnel and between health personnel and *Hälsotorg* visitors were also covered. The manual observations notes, impromptu conversations and personal reflections were recorded in field notes. The notes were then expanded when the situation allowed or at the end of the day to identify assumptions, make sense of the data, and record personal insights that might have affected the data [34] and discussed with the DN at *Hälsotorg*.

When *Hälsotorg* visitors allowed it, AJM actively participated in the activities which gave the opportunity to closely observe the activity and ask questions in an unobtrusive way [34,35]. Similarly, AJM, helped in the planning of two public lectures during the field study, thus giving insights on how health communication via mass-media was articulated and executed. Field notes were read repeatedly to make sense of the collected data and get a sense of whole. The data was later coded and categorized using qualitative data analysis [34].

### Document studies

Purposive sampling was used to identify documents, printed materials and records [34] that were of importance to health communication and health promotion in PHC. A total of 13 documents and other printed materials used at *Hälsotorg* were identified as crucial to understand how health promotion in PHC was articulated in text and how it is interpreted in praxis as basis to understand the what, how and why of health communication for health promotion practiced in PHC and factors influencing it (Table 1).

The national documents; the public health policy 2007/8:110 and pharmacy (Apoteket AB) Action plan 2002, were identified through an earlier study of *Hälsotorg* implementation analysis [28]. The county council documents were identified during field studies data collection period and obtained through internet searches on the county council website. The rest of the documents included; an evaluation report of *Hälsotorg* in the region, meeting protocols, monthly reports (mainly activities offered and statistics of visitors) kept by all *Hälsotorg* during the field study. All the documents related to the development, visions and goals for health promotion in PHC. Qualitative content analyses were conducted whereby phrases describing health promotion, health communication in PHC as well as PHC’s missions, role and responsibility in health promotion were highlighted and coded [34].

### Focus groups

To explore the knowledge and experiences [34,36,37] of the different actors in the PHC, focus groups were conducted with actors involved in health promotion in PHC (Table 1). Purposive sampling was used to identify potential information rich sources and main actors [37] among health care personnel in PHC and local community members. To gain a better understanding of health communication for health promotion in PHC and capture perspectives and experiences of the different actors who affect or are affected by it, effort was made to include providers, receivers and decision makers of health communication in PHC.

Focus group participants were recruited using snowball methods [38] where PHC manager and DN in *Hälsotorg* played a key role in identifying and recruiting of informants. A letter containing project information and a request for participation was sent out to prospective informants in PHC and to a Swedish language class for immigrants. Respondents to the letter, were later contacted to decide on dates and places for focus groups. Five focus group interviews were conducted. Group 1 and 2 consisted of DNs in PHC (n=9). Group 3, was Hälsotorg’s network (n=10) consisting of PHC managers, a pharmacy manager, dental clinic manager, psychiatric clinic manager, *Hälsotorgs* personnel across the region, and a public health strategist. Group 4 consisted of immigrants from a Swedish language school while group 5 was made up of *Hälsotorgs’* personnel in the PHC of this case study. The total number participants in focus groups was 30 (Table 1).

The immigrant group was a strategic choice as *Hälsotorg* personnel recounted that from their experience, immigrant groups had low health literacy and were hard to reach. During the period of this study, Hälsotorg had contact with immigrants in the Swedish language instruction school (SFI). The immigrants were informed about the study and requested to participate.

Data was collected using semi-structured, open ended interview guide [34,39] divided in two parts. The first part pertained informants’ personal experiences of designing, delivering / receiving health information/ health communication in or from PHC. The second part concerned informants’ knowledge and experience of ICT supported tools for health information and suggestions for improvements of health communications for health promotion. The interview guide was modified to adapt to the different groups of informants in order to capture the varying perspectives, experiences, roles and needs. Focus groups with health personnel were conducted in private rooms within the PHC, while focus group with the immigrant group was conducted in their classroom which was a familiar environment [31]. AJM functioned as the principle moderator in all the focus groups assisted by EO who took notes. A post meeting analysis of the session was held by the researchers at the end of every session to compare notes and identify new ideas (if any) that needed to be explored in the next focus group [37]. Focus groups discussions were audio taped and transcribed per verbatim [34]. Data was read repeatedly to achieve immersion and obtain a sense of whole, then coded and categorized using inductive qualitative content analysis [34].

### Data analysis

Data from focus groups, participatory observations and document analysis were analyzed, coded and categorized separately using inductive qualitative content analysis [34]. Emerging categories from the different data sets were constantly compared to each other and integrated into themes (Table 2) to form a rich description of the case [32]. Coding was initially done by AJM and thereafter negotiated and checked for comprehension with the other co-authors. The derived results were then presented to the DN in *Hälsotorg* for validation. Two main themes emerged from the data analysis namely; communicating health and environment for health communication.

**Table 2:** Analysis process and resulting themes

**Subcategories**	**Categories**	**Themes**
Empowerment Behavior change	Strategies for communicating health	
Channels Methods Competencies	Tools for health communication	Communicating Health
Interpersonal Group ICT mediated	Types of health communication	
Organisational positioning Physical positioning	Strategic Positioning	Environment for health communication
Interests Resources Trust	Collaboration	

### Ethical considerations

The informants were informed on the nature of the study, in accordance with the Swedish Ethical Review Act (SFS 2008:192) and informed consent was obtained from participants. Permission to a conduct field study was granted by the PHC manager. One of the main aims of PAR is to create equality between the researcher and research subjects [31] as well as making explicit the researcher’s assumptions, values and motives [40]. To achieve this kind of transparency, AJM kept the participants informed of the project through; talking to the personnel, taking part in workplace meetings and holding debriefing sessions with the other research members to ensure that personal values and motives did not affect the outcome of the study. Debriefing sessions provided useful arena to discuss difficulties caused by AJM’s dual role of a researcher and health worker when actively taking part in the activities in *Hälsotorg*. However, since the participatory element of enquiry was limited to participatory observation, few problems were encountered as the researcher was sensitive to the participants’ wishes [31]. AJM would always seek their permission prior to engaging in any activity. The study was approved by ‘The regional ethical committee for Lund/Malmö region’, at Lund University in Sweden. Diary number 2009/120.

## Results

The overall analysis shows that health communication is an integral part of health promotion practice in *Hälsotorg* and PHC but there was a dearth of consensus among health professionals on what a health promotion approach is, causing discordance in approaches and practices of health communication. Two main themes emerged from the analyzed data: **Communicating health** and **Environment for health communication** (Table 2). The results are presented in these themes with their categories and sub-categories. Quotations are included to illustrate how the interpretation is grounded in the data.

### Communicating health

Communicating health was identified as a major function for PHC by all informants. This theme captures how health was communicated, understood and practiced. Health personnel identified a number of ***strategies*** and ***tools*** used for health communication as well as ***types*** of health communication carried out in PHC.

### Strategies for health communication

This category mirrored two different approaches used by health personnel to accomplish objectives for health communication; *empowerment* and *behavior change strategies*. Empowerment was indicated in the policy documents, and acknowledged by health personnel, as the ultimate goal for health communication in PHC. Field studies and focus groups indicated however that the empowerment strategy was more evident in *Hälsotorg* and in CHS compared to the rest of the PHC units.

In the *empowerment strategy,* health personnel assumed the role of a dialogue partner and facilitator for the learning process of patients and visitors. Decision were made based on the receiver's understanding of the information. This approach was commonly referred to by DNs as ‘m*eeting the clients where they are, in order to guide them to where they want to go in terms of better health*’. In most *Hälsotorg* this empowerment strategy mostly focused on building capacity and providing tools for visitors to make informed decisions or creating solutions to health problems or lifestyle changes through a dialogue, while in CHS, it focused on facilitating empowerment of parents and creating a supportive environment for families. As one *Hälsotorg* visitor expressed:

“Here (in Hälsotorg) I can discuss different things at the same time, I was referred here by my Doctor because of my high cholesterol but then, I ended up discussing my sleep patterns that is more disturbing to me really more than high cholesterol (laughter)…You can’t do that at the PHC” (Hälsotorg visitor 1)."

Or as another informant expressed;

"“That’s how we work all the time, promoting health and preventing ill health in the home now we focus a lot on unhealthy drinking and we routinely ask both mothers and fathers about their drinking habit not just mothers. It is important that children are safe and parents who need help, feel they can get it” (FG 1)."

In contrast to the empowerment strategy, the *behavior change* strategy focused on disease and risk prevention. Health personnel were more or less authoritative and ‘instructed’ the patient/visitor, assuming the role of expert, who ultimately informed the patient /visitor, what was best for them. One of the (health) personnel explained the health communication process as follows:

"“We normally go through their (patients’) eating habits and daily exercises together if any and then I show them what they are doing wrong. Then I “teach them” the right diet and tell them that they have to exercise at least half an hour per day. Some do not follow our advice but that’s their own responsibility” (FG 2)."

Comparison of data from interviews and field studies showed that the different strategies could be traced to health personnel’s understanding of the health promotion concept and the exhibited discrepancy between their intentions to promote health and the existing praxis for health communication in their respective units.

### Tools for health communication

This category included *tools as channels, tools as methods,* and *tools as competencies.*

*Tools as channels* for health communication included telephones, printed and electronic materials, and Internet-based resources. These were used for health communication with patients/clients/visitors separately or in combination, depending on the nature or purpose of the activity and the desired outcome. According to informants and observations, telephone, printed and electronic materials were common channels for health personnel’s communication with patients and visitors. Health personnel used Internet mostly to search for health information for the purpose of updating their knowledge or to retrieve health information materials for their clients/visitors. Patients and visitors used telephones mostly for health communication with health personnel, while Internet was used to seek knowledge in an area of interest or concern;-mainly chronic diseases and self care.

*Tools as methods* included questionnaires, brochures, and electronic or printed health tests*.* Almost all individual counseling sessions were initiated using a printed or electronic health questionnaire followed by a dialogue. Health personnel were positive towards these tools, as they gave structure to health communication activities. However, according to health personnel and visitors these methods could potentially encourage an expert-laymen driven approach, reducing health communication to filling of questionnaires instead of having a dialogue between partners. Health personnel acknowledged the shortcomings of the questionnaires as an effective tool for promoting health as follows:

"“…yaaa (hesitating) …we don’t produce them (questionnaires) ourselves…they are standardized and most people have more than one health concern, there is a risk that you focus too much on the questionnaire instead of listening to the patient” (FG 2)."

*Tools as competencies* for health communication encompassed knowledge, abilities and pedagogical skills for health communication, which were perceived as necessary tools for imparting or working with health promotion. Knowledge and abilities refer to skills necessary for health personnel to impart health related knowledge that influences individual health choices and self-care. Pedagogical skills refer to health personnel’s ability to apply those skills appropriately and in a way that fosters empowerment in their clients/patients. DNs, in particular, expressed a desire for internal courses to improve their pedagogical skills and capacity to act as health promotion agents. As expressed in one of the focus groups:

"“…of course we can be better at communicating when it comes to health promotion and disease prevention…but it is not always easy. For instance, when you get a patient with hypertension who is a bit fat, you can talk about diet…but to apply it generally in the day to day activities is hard..that needs a different kind of structure, skills and knowledge …pedagogical skills that unfortunately are not there in us…” (FG 1)."

### Types of health communication

Three types of health communication were identified from the data: *interpersonal, group* and *ICT mediated* health communication. *Interpersonal communication* was the most common type of communication used in PHC and at *Hälsotorg* as the majority of activities/services targeted individuals. Motivation Interview (MI) was the recommended method for individual health counseling in the county council policy document and also acknowledged and used by the DN’s. Face-face verbal communication between patients/visitors and health personnel in either planned individual counseling or during ‘drop in’ sessions. The patient/visitor’s needs and abilities were the main focus of interpersonal communications. According to health personnel, it is important to identify patient’s source of motivation as opposed to health personnel’s. As exemplified in the following quotation by health personnel:

"“… it is hard for people to change their habits…but we try to help them identify things that would make them want to change, for example if a visitor is diabetic and overweight…to us it is natural to say diabetes is the problem, but maybe the person wants to lose weight because they want to look beautiful…(all informants nod in agreement)…then beauty is that person’s motivation but in the end the results (of losing weight) would be good for their diabetes too” (FG5)."

*Group communication* was mostly used at *Hälsotorgs* during group activities such as physical training and open lectures on different lifestyle related ill health. Different kinds of physical training sessions were offered for example; aerobics for physically challenged persons (including wheelchair- bound persons) and power walking. Open lectures also varied in content, from stress to cardiovascular diseases. These activities paved way for group communication and facilitated dialogue on varied health issues between health personnel and community members.

Findings show that group activities were appreciated by both *Hälsotorg* personnel and visitors. *Hälsotorg* personnel saw these sessions with group discussions, as opportunities to communicate health to a larger population, something that is not always easy to accomplish in the day to day work. For visitors, these sessions were more than just an opportunity to exercise or get health information; they presented an opportunity for collaborative learning and opportunity to act on the knowledge acquired for health gains. This would not have been possible if *Hälsotorg* had not created supportive and inclusive environment for all citizens, regardless of health condition. As expressed by a *Hälsotorg* visitor:

"“Hälsotorg has saved my life…I come every Tuesday and walk with this group…it is nice…I made some friends…and the DN can see when somebody is having difficulties…I have a bad heart and I would never dare go on long walks like this if I didn’t know there was somebody to help me if I collapse…she sometimes tells me and the whole group to reduce our pace…because she “sees” when I am struggling…” (Hälsotorg visitor 6)."

*ICT mediated health communication*, especially the Internet, was regarded as an important media for health communication by all informants. Younger *Hälsotorg* visitors and immigrant informants were more positive to the use of internet as a source of health communication; they reported using Internet for health information needs more extensively than health personnel and older *Hälsotorg* visitors. Younger *Hälsotorg* visitors and immigrants reported using internet to search information on lifestyle related ill health. Mainly information on weight loss, diet, smoking cessation and stress as well as cardio-vascular diseases. Information on how to contact the local PHC clinics and hospitals was also reported. Immigrant informants used both Internet and digital television, as these channels offered health information in their native languages. *Hälsotorg* personnel frequently used web based-lifestyle questionnaire on the Pharmacy’s website *apoteket.se* to tests the visitors’ diet, sleep, exercise, smoking and drinking habits.

Results from the web based-lifestyle questionnaire was used as a basis for individual counseling sessions regardless of what health problem the visitors came in for. A clear irritation was noted among some of the visitors who did not see the connection in for example the hypertension control they came in for and answering the long questionnaire while others appreciated the questionnaire, noting that it has helped them realize that they need to eat better balanced diet or stop smoking for example.

A common phenomenon noted during the field studies was the number of *Hälsotorg* visitors coming in with health information acquired from the Internet, wanting to discuss the content and validity with the personnel. A DN expressed criticism of the Internet as a source for health information as follows:

"“…patients come with all kinds of information, sometimes wrong information and it’s hard to counter that kind of misinformation…the new health channel would be good because we will be able to give them access to health information that we know is correct” (FG 3)."

### Environment for health communication

The environment for health communication was seen as both a facilitator and barrier to health promoting communication efforts in PHC. Two important factors affecting the environment of health communication were identified: ***Strategic positioning*** and ***Collaborating for health communication***. Positioning of *Hälsotorg* within a PHC center affected health communication at the PHC units and *Hälsotorg*, as well as the collaboration efforts between the different actors.

### Strategic positioning

According to the analyzed policy documents, *Hälsotorg* were strategically placed both organizational and physically within the PHC context to provide local citizens with health promotion and disease prevention services; and to help them navigate the health care system using health information and health communication as strategies. Provision of these services was aimed at increasing health literacy and capacity for self-care among the population, which was supposed to reduce pressure on the PHC medical services.

*Organizational and physical* positioning were identified as important factors shaping health communication practice in PHC. *Organizational positioning* referred to the placement of *Hälsotorg* within the PHCadministrative organization. According to the National Pharmacy Action plan, placing *Hälsotorg* within the PHC and the pharmacy organizations was a strategy to profile health promotion and disease prevention services in order to involve local citizens in a health dialogue, help people manage their health problems and stay healthy. The Pharmacy, which already had counseling services and a large flow of mainly healthy customers, could play an important role in promoting health at population level in collaboration with PHC. The county council plans also highlighted the importance of adopting a health promotion approach and the creation of a supportive environment for health within the health care services. *Hälsotorg* was pinpointed as an important setting for realization of these esteemed goals in the first plan (2007–2009) but was not mentioned in the second plan (2008–2010).

PHC was associated with being sick in most people’s minds, according to DNs. ‘Healthy people’ rarely visited PHC, a statement that was echoed by immigrant informants and *Hälsotorg* visitors. They only contacted or visited PHC when they were ill, prior to their knowledge of *Hälsotorg’s* existence. The most frequent visitor was a middle-aged woman or an elderly male pensioner with multi-health problems. Some of the health personnel perceived the clientele as being the ‘wrong type’ for health promotion interventions. They expressed a wish to relocate *Hälsotorg* in order to attract a ‘younger’ and healthier clientele. As expressed below 

"“ It is perhaps about the kind of people who walk through our walls (referring to the PHC building)… am I being mean? It is the wrong target group. I feel like…maybe we ought to go to schools, year 7, 8 9, those are the ones we should be aiming at” (FG 2)."

However, not all health personnel held the same view. Some regarded the placement of *Hälsotorg* within PHC context as perfect as related by other health personnel

"“ …we cannot only target the healthy, we have an obligation to help those who already experience ill health like those with diabetes, they really consume a lot of resources and the best place to “capture” them is in PHC where they come for regular controls. If we can help them prevent further health deterioration like kidney failure, then it is worth the effort” (FG2)."

In ambition to reach out to a larger and ‘different’ audience with health communication, *Hälsotorg* personnel conducted ‘*Hälsotorg* on wheels week’ where they set up camps in the town centre and offered their services to the general public, a move that was much appreciated by both the personnel and the public, according to *Hälsotorg* personnel’s own documentation. The DNs’ opinion about the positioning of *Hälsotorg* was not shared by informants in FG 3, who regarded *Hälsotorg’s* positioning to be the best location to intercept people suffering from minor health problems with services geared towards primary and tertiary disease prevention.

DNs in the focus groups (FG1 and 2), indicated that the organization leadership promoted the image of PHC as a setting for ‘sick care’ through policies on the physical environment of the clinics. An example given by informants was a policy where no posters or information leaflets with health information were allowed in the GP waiting rooms while it was allowed in the CHS and *Hälsotorg.* This differentiation caused frustration among the personnel, as one of them expressed:

"“Sometimes, I feel like we could be more proactive and put up information pamphlets and posters on HEALTH! But no, we are not allowed, no reasons or discussions! ”(FG2)."

Another informant suggested that the PHC management thwarted their efforts to use health communication proactively, expressing disappointment as follows:

"“.we don’t have notice boards here, I tried to put up some notices on health promotion activities but was summoned and told that I cannot do that by the management!…I don’t understand how they reason” (FG 5)."

*Physical positioning* refers to the placement of *Hälsotorg* in the entrance hall of a PHC and/or a Pharmacy or a hospital. Field study observations revealed that *Hälsotorg’s* physical position made it easy for people to stop by and discuss health concerns, obtain help to navigate the health system e.g. to find the appropriate health clinic at which to seek help. On arrival at the *Hälsotorg*, curious passersby and referral patients from PHC were introduced to a variety of free services offered. These included universal health information, individual health counseling and access to trustworthy Internet-based health information sites for *health promotion*.

For visitors with a high risk for lifestyle-related diseases like diabetes and cardiovascular diseases, *disease prevention services* such as hypertension control, lifestyle tests and group physical activities were offered. The most popular group activity was aerobics for people with physical disabilities.

A disadvantage of the openness of *Hälsotorg*, was the surrounding noise and lack of privacy during consultations and individual counseling. This was observed during field studies and later acknowledged by the informants. The noise often led to irritation and disgruntlement, thereby affecting the quality and outcome of the sessions. *Hälsotorg* personnel expressed that the planned *Hälsotorg* channel would partly solve this problem:

"“This virtual Hälsotorg channel can be good for us; it presents a totally new way of planning individual counseling we can offer a quieter, individual based counseling in the comfort of their homes” (FG 3)."

Adding that the privacy presented by the VHT would enable them to increase the range of services offered to their clients as follows:

"“We can even put up programs (in VHT) where clients can work at their own pace and convenience, without stress or worrying about being disturbed” (FG 3)."

### Collaborating for health communication

Collaboration within and outside the health care services such as NGO’s, churches, local communities and municipalities was highlighted as very important for promoting health and providing a supportive environment for health (County Council plan 2007–2009). *Hälsotorg* was specifically pointed out as a significant converging arena for the different actors to collaborate in creating a supportive environment to achieve health services’ health promotion goals, a setting for communicating health with both patients and local citizens (ibid).

Locating *Hälsotorg* within the organizational and physical boundaries of health care services resulted in successful collaboration between different professionals and health care organizations for many years, according to the informants and document analysis. Informants acknowledged that making use of the available resources within the different sections of the PHC organization would benefit patients/visitors especially, in health services where lack of resources and time constraints was the norm. However, different structural and organizational factors served as facilitators or obstacles to collaboration efforts. Three categories; *interests*, *resources* and *trust* were identified as factors affecting collaboration efforts and thereby health communication for health promotion purposes.

Collaboration between organizations/professions depended on shared common *interest* in terms of either the same target group and***/***or similar organizational demands. PHC organization in this study was made up of specialized units; CHS, GP and DN consultation. Each unit was allocated resources to work with specific or prioritized target groups. *Hälsotorg* personnel expressed a feeling of marginalization, which they attributed to the fact that they targeted ‘healthy clients’ as opposed to sick/ill patients targeted by the other PHC units. During the field study it was noted that *Hälsotorg* personnel unsuccessfully tried to enlist the help of DNs with special competencies such as diabetes or incontinence, to give a public lecture at *Hälsotorg*. Promoting health was conceived as ‘non-urgent’ and was not prioritized, which explained the difficulty of establishing collaboration with *Hälsotorg*.

Organizational demands of “need-based” prioritization resulted in prioritization of curative and risk-disease prevention in most PHC units. External organizational demands such as national directives and policies were also cited by health personnel as factors affecting interests and, thereby collaboration. For example prioritization of child and geriatric health in the policy years 2008–2010, led to PHC units prioritizing collaboration around these two target groups. Since Hälsotorg did not have a specified ‘target group,’ it experienced difficulties finding collaborating partners in PHC. In an effort to bridge the gap between *Hälsotorg* and the other PHC units, all the hypertension controls were relocated to *Hälsotorg*. This was a decision that was not popular among *Hälsotorg* personnel as it was seen as ‘medicalization’, of their services, as expressed below:

"“…it undermines the whole purpose of my work…I don’t mind them coming but I have to document in their medical journal…I have to talk about their medical history, diseases…that becomes the focus!…Hälsotorg becomes the extended arm of their medical clinic..” (FG5)."

Availability of resources was identified as pre-requisite for communicating health to the public. However, resources were scarce in PHC according to the health personnel. Thus lack of or poor collaboration between different professions and organizations was attributed by the DNs to the scarce resources. Two types of resources were identified from the data: time and economy. Lack of time was attributed to a high workload and little time allocated to each patient, often ageing and multi-morbid patients. However, some DNs suggested that unwillingness to think ‘outside the box’ and negative attitudes towards collaboration more than workload contributed to poor collaboration. Lack of economic resources was also cited by health personnel as a hindrance towards engaging in activities outside the prioritized areas. Health personnel pointed out that they operated on a tight budget, with constant cutbacks which forced them to focus on ‘their’ areas of responsibility.

*Trust* was identified as an important collaboration factor in and for health communication between health personnel and visitors; and between health personnel in different PHC units. *Hälsotorg* visitors related that they came to *Hälsotorg* and took part in the activities because they had confidence in the professionals who worked there. The information they received was perceived as trustworthy, correct and evidence based as it came from a health care authority. DNs in other PHC units also expressed that it was easier to collaborate with *Hälsotorg* when it was managed by ‘one of them’, meaning a DN

"“…We try to refer our patients to Hälsotorg they are not used to it but we explain that it is one of our own that will help them and the only difference is that there are no medical records. Once they hear they’ll meet a District Nurse, they go willingly…” (DN 8)."

The planned VHT was regarded as an opportunity to overcome some of the collaboration obstacles faced by health personnel. According to health personnel, VHT could be a converging “virtual space” where PHC units could work together but at the same time profile their specific services and communicate with respective target groups.

## Discussion

The aim of this study was to gain a better understanding of health communication for health promotion and factors affecting such communication in a PHC setting, as a first phase for developing the ‘Virtual *Hälsotorg’ (VHT),* an interactive health channel. According to Kreps [8], understanding the context is central to planning of health communication interventions, especially within the health care services, where a myriad of individual, organizational and societal factors influence health related decisions and practice. Findings from this study highlight the interrelation between individual and organizational factors, tools and strategies that affect framing of health communication and, how health communication is communicated, received and understood. These factors need to be addressed by researchers and PHC actors in the planning and designing an ICT mediated health channel for health promotion [8,24], to achieve its goal of improving health literacy [4,14], and to realize the national public health goal of re-orienting health care services into a more health promoting services [18].

PHC in this study is expected to act as a single organization; working towards the same goal of preventing diseases and promoting health for individuals and the community, according to the health policy documents. However, analyses show that the studied PHC faces challenges of catering for a clientele of different ages and health status, as well as serving both individuals and the community as a group. Furthermore, the PHC units were assigned different target groups and adopted different strategies for health communication, making it difficult to achieve the cohesive organization and stated goals. This study therefore highlights a discrepancy between what is stated in policy documents and expressed intentions by health personnel, from the health communication in practice at the PHC.

Collaboration between different actors within and outside the health care settings is an important principle in health promotion. to increase effectiveness and validity of programs [41]. Division of the PHC into specialized units, each with a given target group, ear marked resources for the target group and prioritization were important factors in contributing to the poor adaptation of a health promotion approach in PHC. This demarcation affected content of, and approaches to health communication as well as collaboration between the different PHC units and other partners. Similar results were reported in Johansson et al. [42], where health personnel exhibited both the will and skills for promoting health but lacked the chance to implement them due to perceived lack of opportunity or support from the organization. Thus, organizational structures play an important role in creating a supportive environment to enable integration of health promotion [43]. Health promotion in the PHC studied was regarded as a non-urgent service and as such was not prioritized, which confirms findings from earlier studies showing that health promotion in PHC is sidelined from the rest of PHC activities [42-44].

Health personnel in PHC possess competencies of working with a range of strategies, tools and types of health communication; competencies that could contribute to better ICT based health communication channels such as the planned VHT. DNs in this study have experience of, and skill for working with individual counseling, knowledge and experience that can be used to inform the design of interactive services of the VHT channel; such as tailoring of health information to better suit the intended end users. Tailoring of health information is believed to be one of the most effective strategies for health promotion and lifestyle-changing interventions [23,45,46].

The results also revealed a need for skills development in health promotion approach among health personnel in this study. Majority of informants equated health promotion to primary prevention, disease prevention and/or prevention of risk for diseases. Prevention was the dominant approach in health communication strategies and health professionals’ repertoire. This despite policy documents clearly stated the need for a health promotion approach in PHC and *Hälsotorg* even when working with primary, secondary and tertiary disease prevention. Similarly, health promotion was understood as *activities to promote health* as opposed to *an approach to health promotion*. According to Irvine [47], health professionals in primary care settings, including nurses, lack adequate knowledge to integrate health promotion in their daily work in an effective and planned manner. Thus there is a need to prioritize education and training of health personnel in health promotion knowledge and skills. By involving them directly in the development process of the planned health communication channel, collaborative learning could be facilitated through dialogue between different professionals and lay people.

Allocating *Hälsotorg* within the PHC context resulted in a symbiotic relationship between *Hälsotorg and PHC. Hälsotorg* contributed to a more health promoting PHC services through its health promotion activities while PHC’s narrow and “reactive” prevention approach were forced upon *Hälsotorg* despite protests from *Hälsotorg* personnel*,* like the hypertension controls. However, results also show that *Hälsotorg* and PHC collaborated in the planning and hosting of theme weeks and public lectures despite their differences. Establishment of VHT could benefit from this existing mutual relationship as it aims to promote health by providing accessible and empowering health communication, and creating a supportive environment for health for individuals and the community. VHT could be a potential and ideal converging point for PHC and *Hälsotorgs’* health promotion and prevention approaches. This collaboration could further strengthen the PHC’s health promotion ambitions as stated in the policy documents.

DN’s in this study blamed the poor adaptation of health promotion approach in PHC to the lack of support and interest from the management. Similar results were displayed in Johansson et al. study [42], where health personnel had both the will and skills but lacked the chance to show them due to perceived lack of opportunity or of support from the organization. In this study however, there seems to be contradictions, as participatory observations and meetings with the PHC leadership revealed a willingness among PHC leaders to create infrastructures to improve health communication for the purpose of promoting health. These different perceptions could be the result of the lack of dialogue between PHC leadership and DNs.

According to previous studies [19,45,48], trust can be a defining factor for health information seekers**’** use or rejection of the content of health information on the internet. Trust in content and professions were also cited as two most important factors for choosing health communication resources by local citizens in this study. Pilemalm et al. [45] suggest that involving end users in the design process increases trust among them and thereby probability of their using the system. There is therefore a need to involve all the actors; from PHC managers to DNs in a dialogue during the process of developing VHT; in order to create trust between PHC actors, facilitate sense of shared ownership and sustainability [45,49].

Communicating health is given as an important function in PHC however; results show that there was a lack of synthesis in approaches, strategies and tools to achieve this common goal of promoting health and preventing diseases at individual and community levels. Similarly, empowerment was stated as the ultimate goal of health communication initiatives in PHC but results show that behavior change was the most common approach. Earlier studies have shown that health communication for the purpose of promoting health within health care services, lack a broad socio-ecological health promotion approach [8]. An approach that is necessary to increase individual and population health literacy in order to tackle the determinants of health and the growing burden of chronic diseases [4,6,8]. In order to identify a common health promoting approach and strategies based on health promotion values and principles, a participatory design involving both end users and providers throughout the design process will be used. Participatory design is attributed to contribute to capacity building as participants learn with and from each other while working towards the same goal, making it an appropriate method for development of VHT [24,45].

Data analysis revealed that PHC personnel face a growing challenge of addressing health queries from informed patients and visitors who are more versed with internet use than themselves. In order to meet this, and other future health communication challenges, health personnel need to improve their capacity for using internet-based information [19,50]. Lack of health information in other languages, besides Swedish, is another aspect that needs to be taken into consideration as studies indicate that immigrants generally experience poorer health than native Swedes [43]. According to the Swedish board of statistics, immigrant communities in Sweden increased from 95750 in 2006, to 96467 in 2011. Prognoses indicate that this trend will continue [51]. An accessible Internet-based health communication could be a strong motivation for immigrants to seek health information frequently and manage their own health. One of the major challenges to introducing a new technology in PHC is the need to increase the capacity of health personnel’s ability to use ICT resources effectively while paying attention to the eminent risk for contributing to communication inequalities and digital divide [19]. Equity and inclusion of the needs of non- Swedish speakers will need to be considered by enabling participation of these groups in the design process of health promoting services.

### Study strengths and limitations

Use of triangulation of methods and involving other researchers and informants in the data analysis process provided a rich description of the case and context. Furthermore, this study revealed that a multi-method approach unearths more details that are difficult to identify using a single method, for instance, the discrepancy between policy and what is practiced. This provides readers with information to make their own judgments on the study’s applicability in similar contexts, thereby increasing the study’s transferability [52].

Prolonged participatory observation of three months increased the study’s credibility [53] and enabled the researcher to study not only what was present but also what was ‘missing’. Two important observations made were; the lack of communication between PHC and *Hälsotorg* personnel and absence of pharmacy personnel at Hälsotorg [34]. Participatory observations also gave a detailed documentation of the methodology used for health communication and transparency of decisions, which increases the dependability of this study [52].

By familiarizing with the target groups, the researcher also gained ‘access’ to the field as well as an opportunity to recruit participants for the continued VHT project. According to Smith et al. [40], the success of a PAR research project, like the VHT, depends upon the establishment of an environment for trust between the researcher and the subjects of the study. Furthermore, this phase resonated well with the ‘listen’ phase of the STAR model [27] which entails interacting with the target groups, familiarizing with the context, identifying how target groups interact with technology and carrying out a needs assessment.

A limitation of the study is that it is built on one *Hälsotorg* and one PHC, and as such, based on a small number of informants. This may have had an impact on the results, as the experiences of the other *Hälsotorg* have not been explored fully.

Confining the field study to only one *Hälsotorg* may have narrowed the results as a previous study [28] showed that *Hälsotor*g offer different services and some had existed longer than others. However, expanding the case to include workers from the other *Hälsotorg*, was an effort made in order to compensate for the above mentioned limitations.

Exclusion of GP’s and other health professionals, like dieticians and physiotherapists, from the study is a shortcoming as they could have contributed with valuable information to the study. However DNs, included in this study, was the professional group in PHC who were responsible for health promotion services. Including GP’s was considered, but was not feasible as a majority of the GP’s working at the PHC, at the time of the study, were hired on temporary assignment basis.

## Conclusions

This study identified challenges facing the development of health communication for health promotion in PHC. Understanding the opportunities and obstacles for health promotion and health communication in PHC makes it possible to start a dialogue with the different actors identified in the study i.e. health care personnel, PHC managers and local citizens. Engaging the actors in a dialogue could facilitate a consensus on common strategies to overcome the hindering factors and capitalize on the opportunities.

The most significant challenge in developing an ICT supported health communication channel for health promotion identified in this study is profiling a health promotion approach in PHC. To achieve VHT’s health promotion intentions, the development of VHT channel will have to be based on health promotion values and principles of empowerment, participation, holistic and intersectoral approach, equity, sustainability and multi-strategy. There is a need for a shift of focus from individual to a more population- based orientation, placing emphasis not only on people at risk but also directed at health determinants [22,23,25]. Furthermore, there is a need for a combination of different strategies, aiming at effective participation of all stakeholders on equal terms, and on professionals taking an enabling role instead of an expert role when communicating with patients/PHC visitors [8,23,45]. Finally equity issues need to be addressed through the creation of accessible health communication to improve health literacy [14], even for non- Swedish speakers as well as those with low literacy [53]. By addressing these factors in the design of e-Health services, health communication via an ICT supported channel could be health communication for promoting health, i.e. ‘health promoting communication’.

Although this study provides valuable insights to factors that need to be taken into consideration prior to development of an ICT supported health channel, there is a need for further research to better understand the needs for health communication among non-Swedish speakers and to further explore the relationship between the different organizational and social factors affecting health communication.

## Abbreviation

ICT: Information Communication Technology; PHC: Primary Health Care; VHT: Virtuellt Hälsotorg’ (Virtual Health Channel); WHO: World Health Organization; STAR: Spiral Technology Action Research; PAR: Participatory Action Research; GP: General Practitioner; DN: District Nurse; CHS: Children Health Services.

## Competing interests

Authors declare no competing interests.

## Authors’ contributions

AJM, EO and BH contributed to the conceptualization and design of the study. AJM conducted data collection, analysis and drafting of the manuscript. AJM, EO, SE and BH contributed to interpretation of the results and critical revision of the manuscript. All authors have read and approved the final manuscript.

## Pre-publication history

The pre-publication history for this paper can be accessed here:

http://www.biomedcentral.com/1472-6947/13/17/prepub
